# Quick Annotator: an open‐source digital pathology based rapid image annotation tool

**DOI:** 10.1002/cjp2.229

**Published:** 2021-07-19

**Authors:** Runtian Miao, Robert Toth, Yu Zhou, Anant Madabhushi, Andrew Janowczyk

**Affiliations:** ^1^ Department of Biomedical Engineering Case Western Reserve University Cleveland OH USA; ^2^ Toth Technology LLC Dover NJ USA; ^3^ Louis Stokes Veterans Administration Medical Center Cleveland OH USA; ^4^ Precision Oncology Center Lausanne University Hospital Lausanne Switzerland

**Keywords:** digital pathology, computational pathology, deep learning, active learning, annotations, open‐source tool, nuclei, epithelium, tubules, efficiency

## Abstract

Image‐based biomarker discovery typically requires accurate segmentation of histologic structures (e.g. cell nuclei, tubules, and epithelial regions) in digital pathology whole slide images (WSIs). Unfortunately, annotating each structure of interest is laborious and often intractable even in moderately sized cohorts. Here, we present an open‐source tool, Quick Annotator (QA), designed to improve annotation efficiency of histologic structures by orders of magnitude. While the user annotates regions of interest (ROIs) via an intuitive web interface, a deep learning (DL) model is concurrently optimized using these annotations and applied to the ROI. The user iteratively reviews DL results to either (1) accept accurately annotated regions or (2) correct erroneously segmented structures to improve subsequent model suggestions, before transitioning to other ROIs. We demonstrate the effectiveness of QA over comparable manual efforts via three use cases. These include annotating (1) 337,386 nuclei in 5 pancreatic WSIs, (2) 5,692 tubules in 10 colorectal WSIs, and (3) 14,187 regions of epithelium in 10 breast WSIs. Efficiency gains in terms of annotations per second of 102×, 9×, and 39× were, respectively, witnessed while retaining *f*‐scores >0.95, suggesting that QA may be a valuable tool for efficiently fully annotating WSIs employed in downstream biomarker studies.

## Introduction

The discovery of biomarkers associated with diagnosis, prognosis, and therapy response from digital pathology whole slide images (WSIs) often requires extracting features from precise segmentations of the histologic structures contained within them (e.g. cell nuclei boundaries, tubule shapes, and regions of epithelium) [1, 2, 3, 4]. Manually annotating each instance of these histologic structures rapidly becomes intractable, even in small cohorts. For example, the number of nuclei in a single WSI can order into the hundreds of thousands, making accurately individually annotating each cell unfeasible. While a number of image analysis based algorithms have been proposed to help reduce annotation effort, they are not yet integrated into tools with user interfaces enabling their employment [5, 6, 7, 8]. Other efforts have resulted in proprietary closed‐source tools [9, 10] which can be too costly to purchase in academic settings, or do not provide an open environment for facile testing and integration of new algorithms. Other approaches provide command line scripts [11] which are not readily employable by lay users, while additionally requiring the colocation of the user, data, and compute‐infrastructure. It is thus clear that an all‐inclusive computational tool designed for remote access by pathologists to aid in this annotation process at scale is needed.

Recognizing the need for a modular, user‐friendly, annotation tool which significantly accelerates annotation tasks, we present here an open‐source image annotation application, Quick Annotator (QA). QA is able to provide significant improvements in annotation efficiency by intelligent usage of deep learning (DL), a form of supervised machine learning which involves multiple neural network layers. QA involves integrating DL [12] with active learning [13], an interactive supervised approach for training machine learning approaches based off selective user feedback. As the user annotates structures in the web‐browser based frontend, a popular DL model (u‐net [14]) is trained in the backend. This DL model then makes predictions highlighting the structure, allowing the user to either accept or refine pixel‐level boundaries in a rapid fashion. This approach allows the DL model to provide feedback to the user, accentuating regions in the image which require additional user input to maximally improve the performance of the next iteration of the supervised classifier. Through this iterative active learning‐based process, QA empowers the end user to spend more time efficiently verifying, as opposed to painstakingly annotating histologic structures.

To aid in the annotation process, a number of common image annotation tools are provided, such as brushes and erasers of various sizes, along with polygon style annotation tools. More interestingly, QA also provides the option of highlighting image regions via the selection of DL‐derived superpixels [15], which are incrementally improved as the DL model improves, facilitating high‐fidelity pixel‐level boundary selection (see supplementary material, Figure S1 and Section SM1). Importantly, QA is designed in an especially modular way such that, as improvements in both DL technology and architectures are discovered, they can rapidly be integrated into QA with minimal modifications to the base application. The main output from QA consists of the binary masks produced by the user in concert with QA. These masks can immediately be used for the computation of statistics or in downstream applications such as feature extraction or training a larger more sophisticated DL segmentation model. Image‐level and project‐level statistics related to the number of annotated objects and regions are also available for review and download. In this work, we demonstrate the utility of QA for segmentation at three scale lengths typical in computational pathology (Table 1 and supplementary material, Figure S2). At the lowest length scale, from five WSIs corresponding to pancreatic cancer, 337,386 nuclei were segmented. For the intermediate scale, from 10 WSIs containing colorectal cancer, 5,692 tubules were segmented. Lastly, for the largest scale, 14,187 regions of epithelium, totaling an area of 35,844,637 pixels, were segmented in 10 WSIs.

**Table 1:** Description of the datasets used for validation of QA along with the demonstrated speedup.

Tissue scale	Histologic structure	Number of slides	Number of ROIs	Number of histologic structures	QA total time (min)	QA human time (QA_t_, min)	Manual time (*M*_t_, min)	Speed up (*θ*_t_)	*f*‐score
Small	Cell nuclei	5	400	337,386	473	391	40,165	102×	0.97
Medium	Tubules	10	100	5,692	121	101	923	9×	0.95
Large	Epithelium	10	100	14,187	167	113	4,433	39×	0.89

As mentioned above, the difference between QA total time and QA human time is that human time removes DL training time, as the human annotator was dismissed to perform other non‐related annotation tasks. On the other hand, QA total time includes model training time under the assumption that the user kept annotating during backend training. Manual time is derived by extrapolating the measured annotations per minute from a subset of the annotations.

## Materials and methods

**Figure 1:**
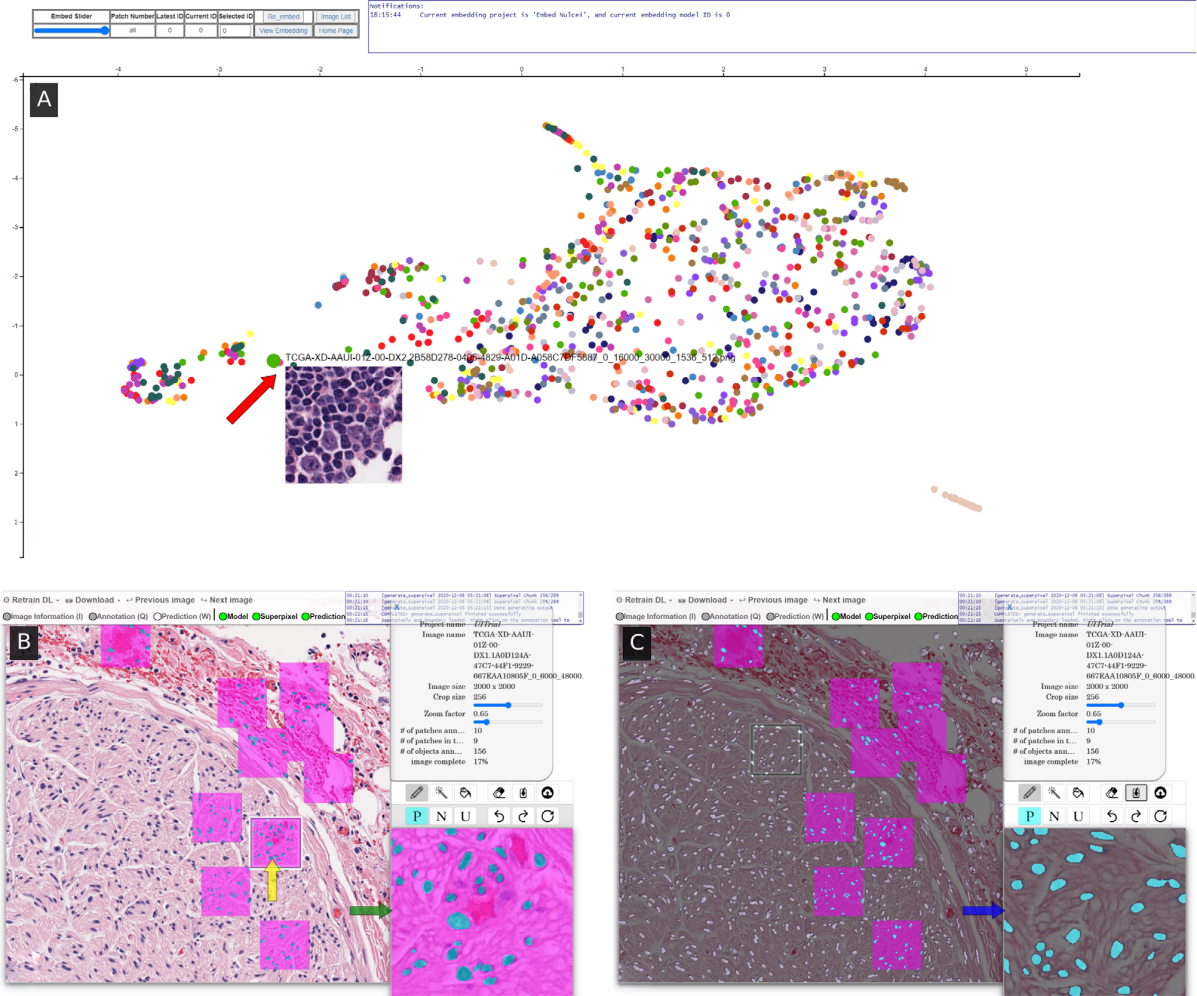
The user interface consists of (A) an embedding plot page, and (B & C) an annotation page. The embedding page is a two‐dimensional representation of all patches in the system, where patches perceived to be similar by the model are plotted closely together, and updates as the model trains. By actively annotating patches across the space, the user provides more diverse training exemplars to the DL model, thus facilitating the creation of a more robust model sooner. When hovering over a dot, a preview of the patch is shown (red arrow), and subsequently clicking on the dot takes the user to the (B) annotation page centered around that patch. There, the user selects (yellow arrow) a square region which is then loaded into the high‐magnification annotation window (green arrow). The user annotates positive regions in turquoise and nontarget areas in fuchsia using common image markup tools. After annotating at least three patches, the user can train a DL classifier to generate annotation suggestions in (C) white overlay. The user may then import the classifier's suggestions into an annotation window (blue arrow) and edit, if needed, before accepting.

In accordance with the QA workflow (see supplementary material, Section SM2), each WSI was broken into tiles and processed individually. To begin, tiles originating from the same WSI were uploaded into QA, which divided these tiles into smaller 256 × 256 patches. A u‐net consisting of a block depth of five layers and 113,306 parameters was trained on these image patches in an autoencoding fashion to produce a baseline model, a process shown to learn features associated with tissue presentation [16]. This base model is subsequently fine‐tuned in a supervised fashion to segment the structure of interest, a common approach to help reduce annotated data requirements [17]. Next, the user viewed all patches processed by this model in a uniform manifold approximation and projection (UMAP [18]) plot (Figure 1A). This plot maps the high‐dimensional space learned by the DL model into a two‐dimensional representation such that patches perceived to be similar by the model are plotted proximally. The user selects dispersed patches for annotation (see supplementary material, Figure S3) to improve training set diversity. As the user annotates these patches (Figure 1B), the DL model begins to make suggestions which can be accepted or modified (Figure 1C).

The time to annotate the tiles is recorded to estimate a metric of structures per second, which is reported in both total time and human time. The difference between these metrics is that human time removes the DL model training time, as the human annotator can be dismissed to perform other unrelated tasks.

To form a manual baseline for comparison, an open‐source digital pathology tool, QuPath [19], was employed. QuPath is one of the most widely used open‐source image analysis toolkits used by researchers and pathologists, owing to its highly polished and intuitive interface, cross platform support, and ease of execution of common analytical workflows. In each use case, QuPath was used to annotate a subset of the data, forming the ground truth for comparison. In addition, the manual time needed to annotate this subset was recorded and used to compute an approximate total annotation time (*M*_t_) needed for completion of the entire task. Quantitatively, efficiency improvement was defined as the ratio (*θ*_t_) between *M*_t_ and QA time (QA_t_). Pixel‐level *f*‐scores were reported comparing the masks created in the manually annotated subset of data with that of QA‐aided annotations to ensure comparable annotations were produced.

## Results and discussion

**Figure 2:**
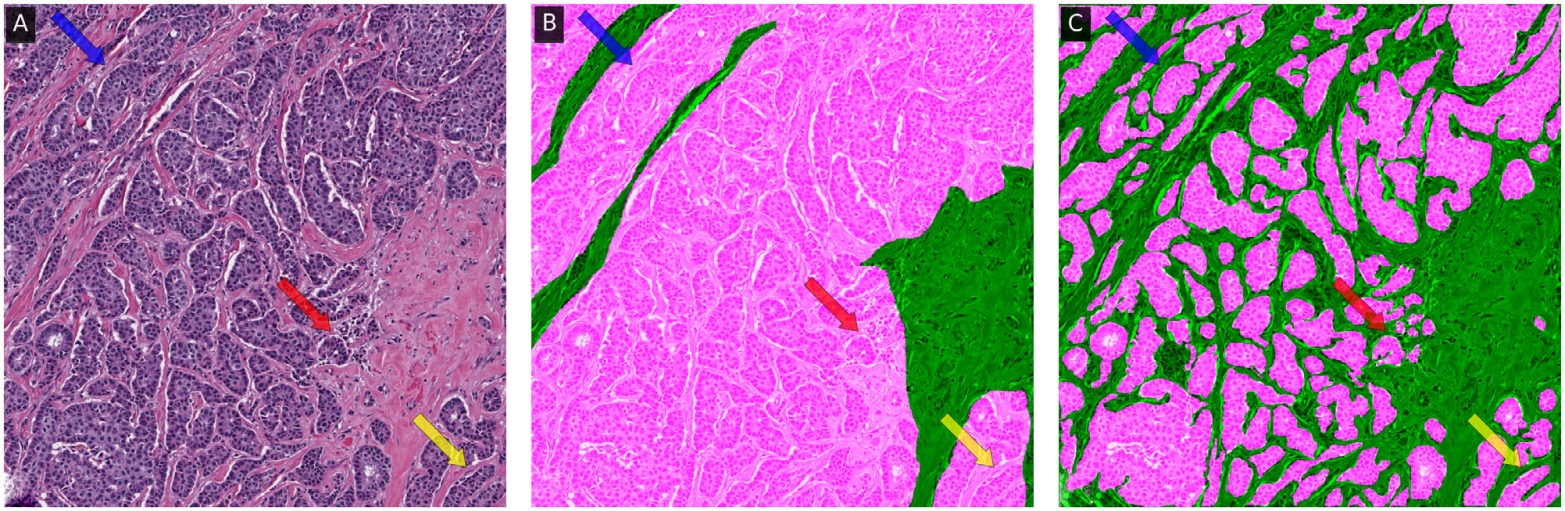
The (A) original 1,000 × 1,000 epithelium ROI with associated (B) manual and (C) QA annotation overlaid in fuchsia, with an *f*‐score of 0.68. In intricate epithelial regions (e.g. areas indicated with arrows), the QA classifier appears to be able to provide annotation suggestions at a level of precision that would not be tractable for a user to perform manually.

Our results (Table 1) indicate that (1) the speed efficiency improvement afforded by QA is significant and (2) QA annotations remained highly concordant with those produced manually. It is important to note that the user is the final arbiter of what is an acceptable annotation, and always has the ability to manually adjust any pixel that they are in disagreement with. Interestingly, differences still remain in the reported *f*‐scores between manual and QA produced masks. This can be attributed to the higher level of precision afforded by computational tools (Figure 2 and supplementary material, Figure S4), a phenomenon that others have reported [12, 20], and which further represents an interesting opportunity for such tools to significantly improve the quality of annotated datasets. Although QA has been validated here using H&E images, given the DL‐based back end, QA is agnostic to stain type and can thus be used with any stain or type of image. The small modifications needed are laid out in the supporting documentation.

**Figure 3:**
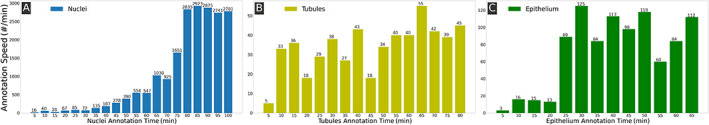
Efficiency metric over time demonstrating the improvement in speed afforded by QA in annotating (A) nuclei, (B) tubules, and (C) epithelium. The *x*‐axis is the human annotation time in minutes and the *y*‐axis is the annotation speed in terms of annotated histologic structures per minute. The trend of performance improvements varies per use case with (A) the nuclei showing a consistent improvement in time, (B) the tubule performance plateauing after annotating a few structures, and (C) the epithelium requiring a number of additional iterations before reaching its plateau. These plateaus indicate the DL model is sufficiently trained to produce suggestions agreeable to the user.

Usage of QA appears to proceed in two distinct workflows. At the beginning, the user is required to provide individual manual annotations, as the model itself is not sufficiently exposed to a representative set of training exemplars. In this workflow, QA and manual annotation efficiency are comparable. This workflow quickly transitions (Figure 3) to one wherein the user effort is more focused on reviewing and accepting predictions from the DL model. Here, the efficiency gains appear to greatly improve, as with a singular click the user can accept large regions containing many structures, as opposed to manually interacting with each of them. This behavior is seen in all use cases, and interestingly creates a point of discussion on the suitability of tasks for QA. In those cases where very few structures of interest are present, such as delineating a single large tumoral region, there is likely minimal value in employing QA, and instead the slides should be annotated manually. On the other hand, when the number of individual annotations required rises, it becomes evident that employing QA may result in significant efficiency gains (see supplementary material, Section SM3).

Worth noting is that, post‐installation of QA, no internet connection is required, thus making it suitable for non‐anonymized clinical data. In fact, given the modestly sized DL networks employed, and its operating system agnostic design, recently purchased laptops are sufficiently powerful to use QA. In spite of this, one can easily host QA on a server with a powerful graphics processing unit (GPU), thus enabling remote access for, e.g., clinical pathologists to collect annotations (i.e. bringing the expert to the data) without the need for the local download and manipulation of large amounts of data (i.e. bringing the data to the expert), an often burdensome paradigm. A helpful consequence of this approach is that no software needs to be installed locally, which is often heavily restricted in clinical environments.

In conclusion, QA is a high‐throughput image annotation tool being publicly released for community review, comment, and usage. QA has demonstrated significant improvements in annotation efficiency, without sacrificing annotation fidelity, and in fact often improves upon what may be possible for humans to complete without computer‐aided tools. Future versions of QA are aimed at incorporating support for directly annotating WSI, as well as further hiding the latency of DL training from the user perspective. The source code of QA is freely available for use, modification, and contribution (https://github.com/choosehappy/QuickAnnotator).

## Supporting information


**Section SM1.** Hyperparameters
**Section SM2.** Experiment setup and workflow
**Section SM3.** Use case‐specific workflows and insights
**Figure S1.** The original 256 × 256 tubules, epithelium, and nuclei ROIs with intensity‐based superpixels and DL‐derived superpixels
**Figure S2.** Original ROIs of pancreatic nuclei, colon tubule, and breast cancer with associated manual annotations and QA annotation
**Figure S3.** Flowchart illustrating the general workflow of QA
**Figure S4.** Demonstration of marked differences in the levels of complexity between less complex and more complex epithelial regions
**Table S1.** Hyperparameters set to different values for different structures (referred to in supplementary material, Section SM1)
