# Supplementary material for: Quick Annotator: an open‐source digital pathology based rapid image annotation tool
Source: J Pathol Clin Res. 2021 Jul 19;7(6):542–7. doi: 10.1002/cjp2.229 (PMC8503896; doi:10.1002/cjp2.229)
Supplement: Supplementary file 1 — Section SM1. Hyperparameters Section SM2. Experiment setup and workflow Section SM3. Use case‐specific workflows and insights Figure S1. The original 256 × 256 tubules, epithelium, and nuclei ROIs with intensity‐based superpixels and DL‐derived superpixels Figure S2. Original ROIs of pancreatic nuclei, colon tubule, and breast cancer with associated manual annotations and QA annotation Figure S3. Flowchart illustrating the general workflow of QA Figure S4. Demonstration of marked differences in the levels of complexity between less complex and more complex epithelial regions Table S1. Hyperparameters set to different values for different structures (referred to in supplementary material, Section SM1) [file CJP2-7-542-s001.pdf]

Quick Annotator: an open-source digital pathology based rapid image annotation tool

R Miao *et al*. *J Pathol Clin Res* DOI: 10.1002/cjp2.229

Supplementary Methods

The reference number refers to the main text list

Section SM 1. Hyperparameters

QA is shipped mostly fully configured and the few hyperparameters of interest are easily modifiable via the configuration file (Table S1). In the use cases discussed here, the hyperparameters requiring modification were those governing the behavior of the superpixels on account of the target structure size. Briefly, a superpixel is defined as a group of adjacent pixels sharing similar characteristics in terms of chromatic, texture, or deep learned feature values[15] (Figure S1). The variable *approxcellsize* is set to the approximate width of the desired superpixel, and works well when set to the approximate width of the structure of interest. The nonnegative *compactness* value determines the regularity of the superpixel boundary, wherein higher compactness encourages superpixels to retain their initial square shape, while lower compactness allows for greater boundary irregularity. As an example, our epithelium use case employed a lower compactness setting due to highly irregular boundaries, versus nuclei which tend to be more consistently circular and thus have a smoother boundary. Lastly, setting a higher *edgeweight* encourages the DL model to focus the loss function on incorrectly classified boundary pixels; increasing this weight is beneficial when clear boundaries are hard to distinguish.

Section SM 2. Experiment setup and workflow

In this paper we focused on 3 histologic structures for segmentation: pancreatic nuclei, colorectal tubules, and breast cancer (see Figure S1).

Each use case followed the workflow presented in Figure S3. However, each use case benefited from slight variations in each step, due to histologic structure size, in order to optimize annotation efficiency (Section SM 3). All experiments were conducted on a Windows 10 desktop with a Nvidia RTX2060 8GB GPU.

Section SM 3. Use case specific workflows and insights

SM 3.1 Nuclei Case

We selected 5 pancreatic cancer WSIs scanned at 40x from TCGA-PAAD dataset verified by Saltz's Group [21]. These 5 WSI images were divided into 2000 x 2000 image tiles. We selected 100 tiles from the generated ROIs. In accordance with the workflow presented in Figure S3, 20 nuclei tiles were uploaded into QA, a u-net autoencoder was trained, and patches were plotted on the embedding plot. Patches were then selected and manually annotated for 5 minutes. The DL prediction model was then trained and predictions were reviewed for modification and acceptance. The process iterated using batches of 20 tiles until all 100 tiles were completed.

In the first 5 minutes of nuclei annotation, even though no DL model was available, QA performed twice as fast as manual segmentation using QuPath [10] due to QA's superpixel functionality (0.27 vs 0.14 nuclei per second). Superpixels enabled one-click selection for a subset of nuclei, notably improving annotation efficiency. As the DL model began to produce better predictions due to more training data, fewer modifications need to be made before accepting the model's proposals. This corresponded to the jump in improvement observed in Figure 3A.

SM 3.2 Tubules Case

We selected 10 colorectal cancer WSIs from TCGA-COAD dataset. These 5 WSI images were divided into 1000 x 1000 image tiles and down sampled to 10x magnification, from which 100 tubule containing tiles were selected. To begin, 20 tiles were uploaded into QA after which the same workflow as to the nuclei use case was employed.

Figure 3B shows the efficiency changes over time as more tubules are annotated and the DL performance improves. Performance fluctuations were the result of differences in quality of WSIs, resulting in some tiles requiring additional correction. The superpixel feature continued to display a one-click selection for many tubules (Figure S1). Compared with nuclei annotation, tubule annotation efficiency converged faster and gave reliable suggestions with fewer annotated patches. The large difference in efficiency performance between nuclei and tubules (103x vs 9x, respectively) resulted from the fact that tubules occupy larger area, and thus there are fewer of them per 1000 x 1000 tile, implying more time is spent transitioning between tiles.

SM 3.3 Epithelium Case

We selected 10 WSIs from an in-house estrogen receptor positive (ER+) breast cancer dataset scanned at 40x, and were processed similar to the tubule use case.

In the first 5 minutes of epithelium annotation, the bulk of the effort was spent manually delineating regions, as superpixel boundaries were not reliable (Figure S1 yellow arrow). This manual process is observed to be slower than the other 2 use cases due to the epithelial compartment's intricate structure. It appears that once a sufficient training set is created, coinciding with 246 annotated regions, the user starts to largely accept the DL suggestions. After this transition point, QA starts to provide improvements in both efficiency and annotation precision. For example, QA was able to provide better pixel-level segmentations in delicate regions which may be intractable for manual annotators (Figure S4).

Supplementary Figures S1 – S4

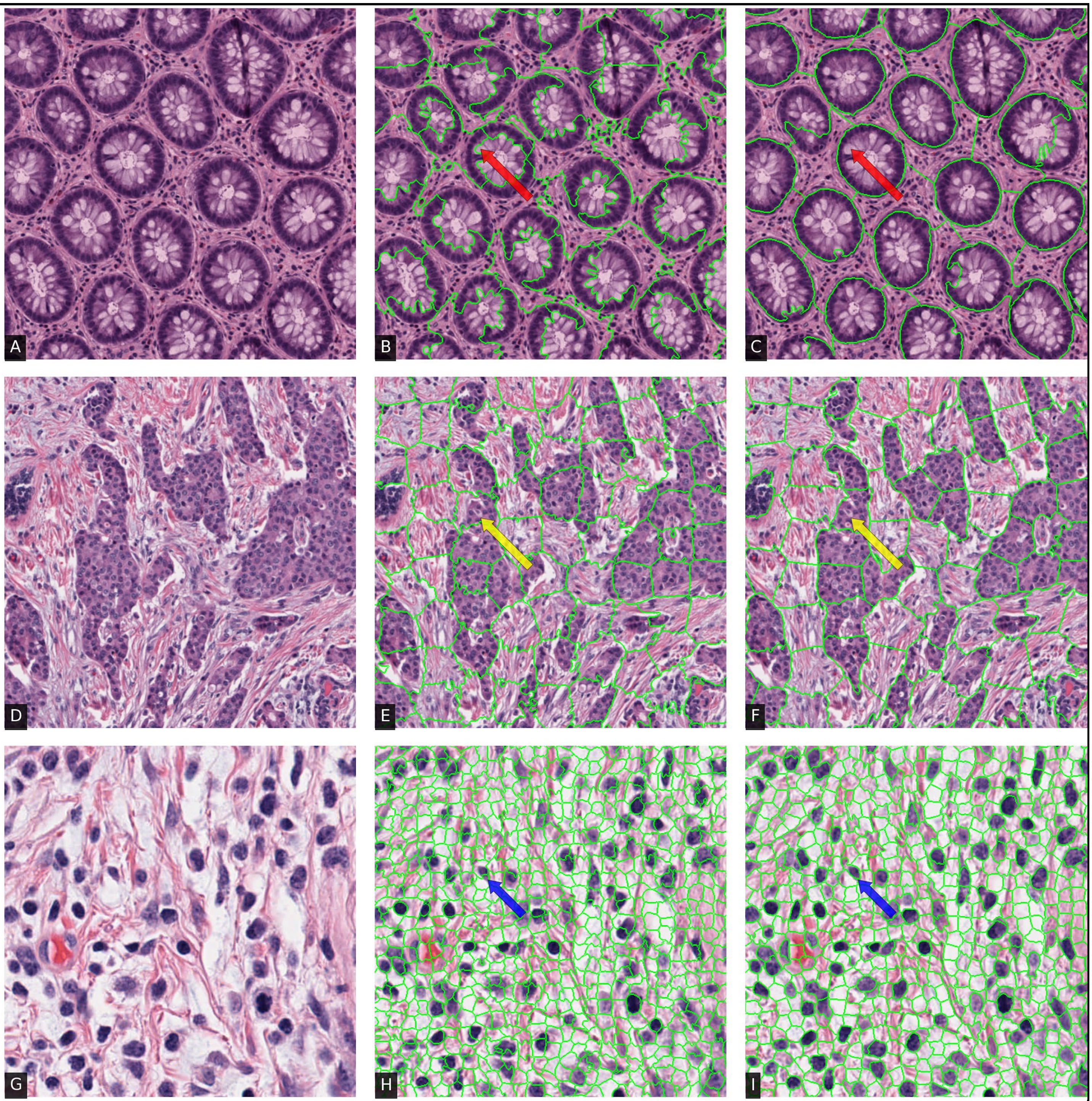

Figure S1. The (A & D & G) original 256 x 256 tubules, epithelium, and nuclei ROIs with (B & E & H) intensity-based superpixels and (C & F & I) deep learning derived superpixels. In QA, selecting a superpixel (boundaries shown in green) results in the groups of connected pixels sharing similar

characteristics to be selected. As the user trains models, the superpixels evolve to capture more accurate boundaries of structures of interest. Later iterations (C & F & I) show increased specificity in terms of hugging structures of interest (e.g., areas indicated with arrows), thus enabling the user to rapidly select structures with high precision while using minimal effort.

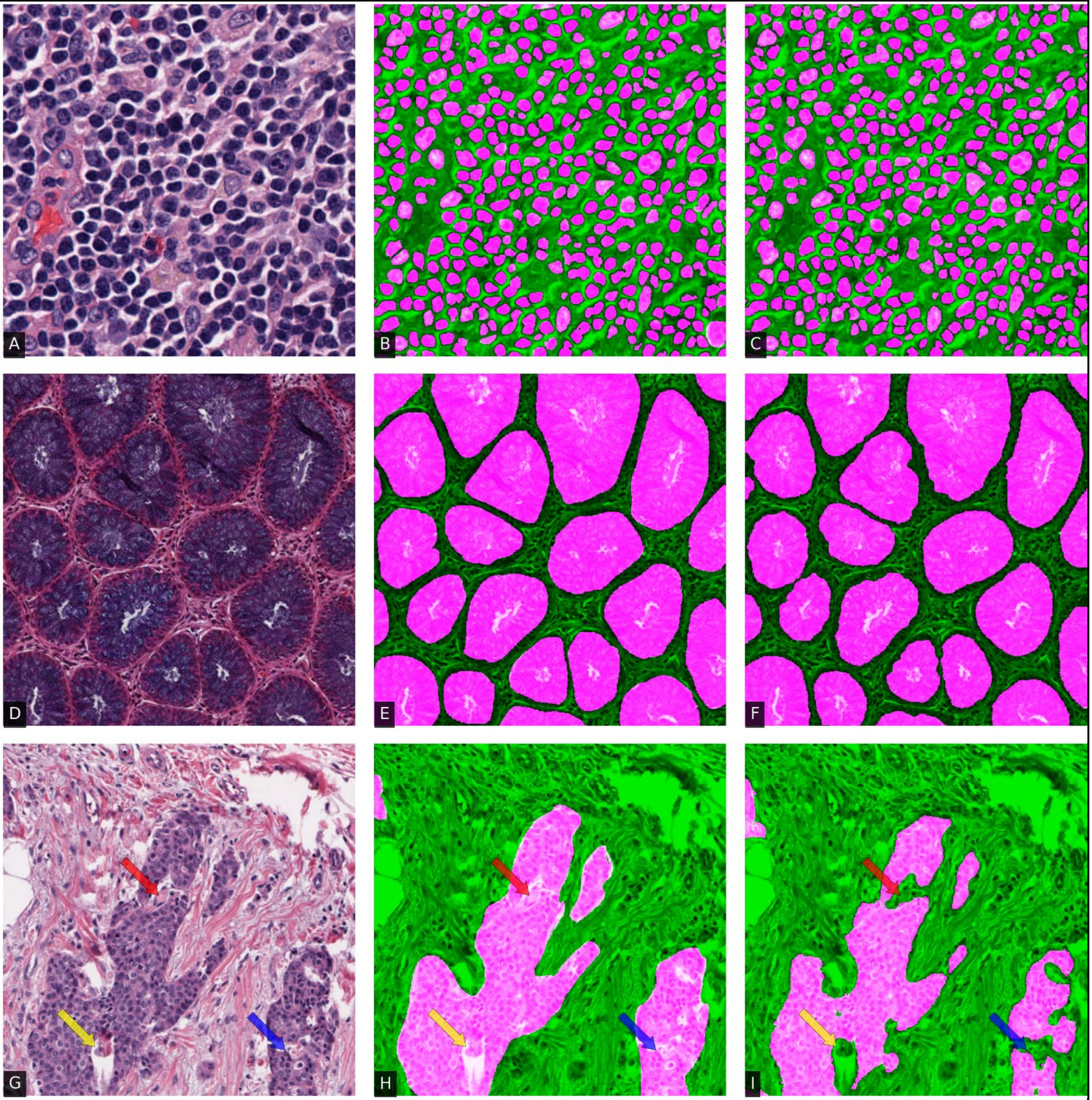

Figure S2. The figure shows original ROIs of (A) pancreatic nuclei, (D) colon tubule, and (G) breast cancer with associated (B & E & H) manual annotations and (C & F & I) QA annotation overlaid in fuchsia. The f-scores of QA versus manual annotation are (top) 0.97, (middle) 0.92, and (bottom) 0.91. In more complex regions (bottom row, areas indicated with arrows), users often produce false positives, likely due to the associated additional time burden needed for intricate annotating.

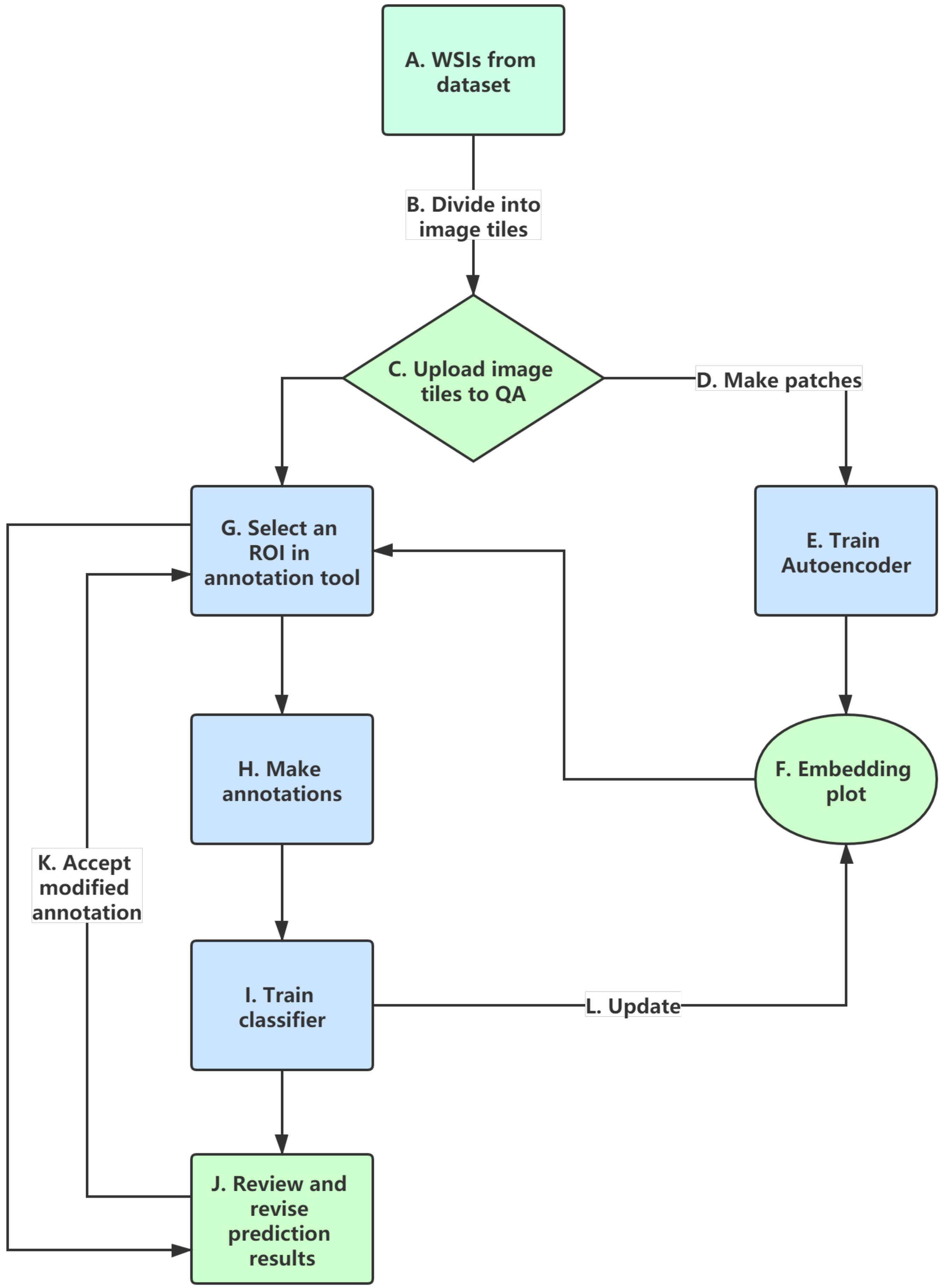
A. WSIs from dataset
B. Divide into image tiles
C. Upload image tiles to QA
D. Make patches
E. Train Autoencoder
F. Embedding plot
G. Select an ROI in annotation tool
H. Make annotations
I. Train classifier
J. Review and revise prediction results
K. Accept modified annotation
L. Update

Figure S3. Flowchart illustrating the general workflow of QA. (A & B & C) WSI are divided into tiles using a provided preprocessing script before being uploaded to QA. (D) Tiles are subsequently sub-divided into smaller patches of size 256 x256 amenable to deep learning. (E) These patches are used to train a u-net in an autoencoding fashion under the guise of initializing the u-net model with dataset-derived weights. (F) Each patch is subsequently embedded into a 2D space using UMAP from a feature vector derived from the transition layer between encoding and decoding of the u-net. (F -> G) The user then views the UMAP embedding plot (Figure 1 A) to select representative patches from the cohort for annotation. (H) Subsequently, the user annotates some suggested patches in the annotations pages (Figure 1B) with annotation aided tools. (I) These annotations were subsequently used to train the u-net and reapply it to the current image tile. (J & K) When viewing the annotation suggestions (Figure 1C), the user may either continue with manual annotation from scratch, or import the DL based suggestions for modification and acceptance of the annotations. (L) The embedding plot can be updated as the model improves, helping to identify poorly represented regions in the training set. As the model provides increasingly reliable suggestions, the user can begin to more confidently accept prediction results (Figure 3), allowing transitions directly from (G) to (J).

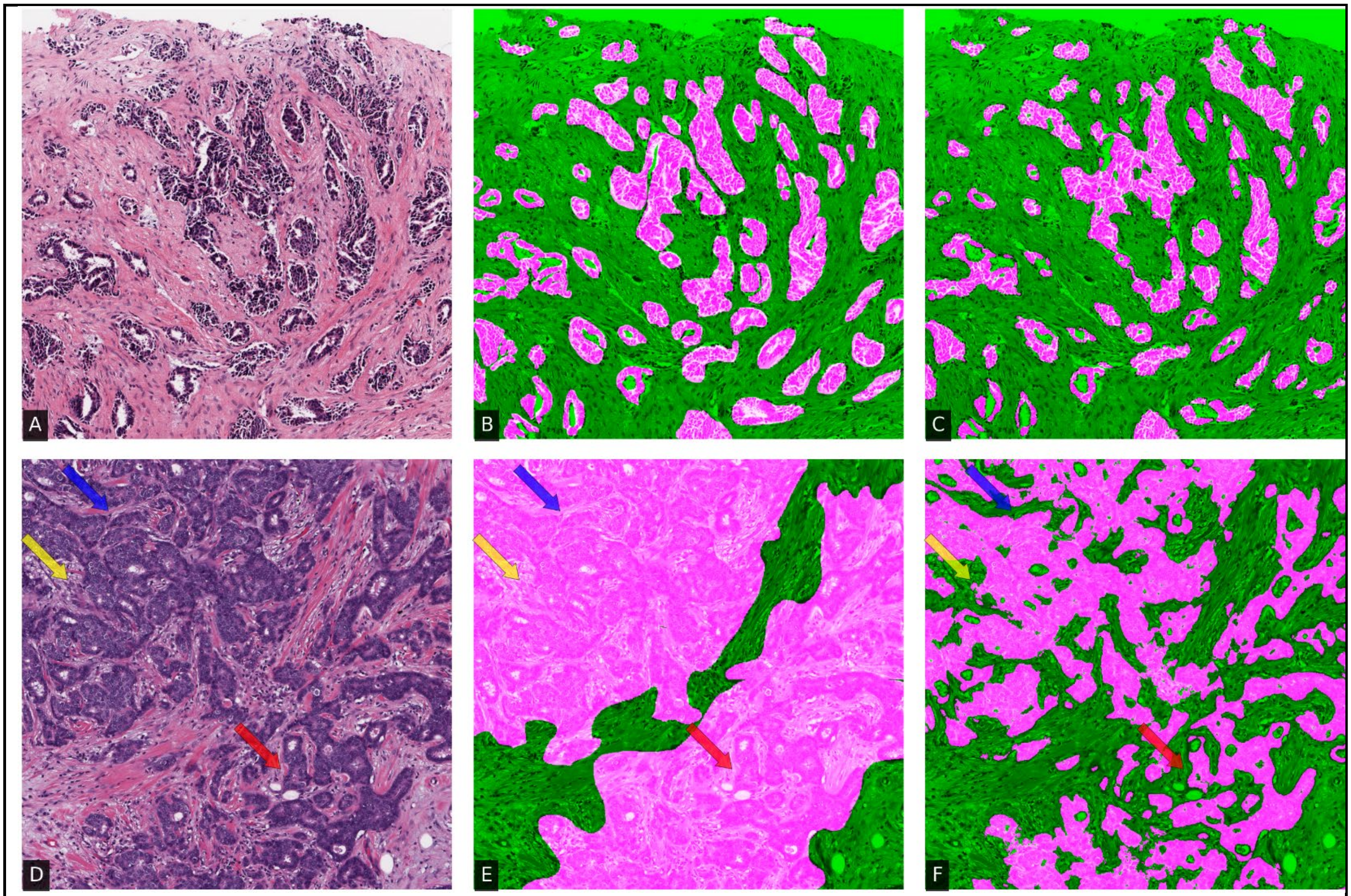

Figure S4. There is marked difference in levels of complexity between (A) less complex and (D) more complex epithelial regions. When manually annotating, large regions are more likely to be marked at a coarse level as indicated in fuchsia overlay (B & E). While QA is able to recapitulate the annotation with high fidelity in less complex images (C, f-score =0.89), in more complex regions (F, e.g., areas indicated with arrows, f-score=0.69) the prediction QA generates appears to be able to provide a level of precision beyond that which would be achievable with human efforts. QA employs an intuitive binary classification such that all pixels are labeled as either epithelial or not epithelial. The latter class includes the totality of all other classes on the image, for example stroma and slide glass.

Supplementary Table

Histologic Structure	edgeweight [train_tl]	approxcellsize [superpixel]	compactness [superpixel]
Cell Nuclei	8	20	10^{-4}
Tubules	2	80	10^{-5}
Epithelium	25	55	10^{-6}

Table S1. Hyper-parameters (*edgeweight*, *approxcellsize*, and *compactness*) are set to different values for different structures.
